# Inoculation with *Trichoderma atroviride* and *T. virens* Induces ROS Overaccumulation and Compromises Pathogen Resistance in *Arabidopsis* 35S::TaEPL1-3 Plants

**DOI:** 10.3390/plants14172794

**Published:** 2025-09-06

**Authors:** Ever Trinidad Astorga-Arzola, Enrique González-Pérez, Alicia Becerra Flora, Juan Francisco Jiménez-Bremont

**Affiliations:** Laboratorio de Biotecnología Molecular de Plantas, División de Biología Molecular, Instituto Potosino de Investigación Científica y Tecnológica, A. C. (IPICYT), San Luis Potosí 78216, Mexico; ever.astorga@ipicyt.edu.mx (E.T.A.-A.);

**Keywords:** cerato-platanins, dual *Trichoderma* interaction, EPL1 elicitor, plant–microbe interactions, ROS balance

## Abstract

Recent studies showed that constitutive expression of the cerato-platanin protein EPL1 from *Trichoderma atroviride* in the *Arabidopsis thaliana* 35S::TaEPL1-3 line promotes plant growth and pathogen resistance. Here, the effect of inoculating this line with *T. atroviride* and *T. virens* on growth and defense responses was evaluated. Inoculated 35S::TaEPL1-3 plantlets exhibited increased fresh weight and more lateral roots compared to uninoculated controls. Infection assays on 28-day-old 35S::TaEPL1-3 and Col-0 (WT) leaves (pre-inoculated at 15 days with *T. atroviride*, *T. virens*, or both) revealed that dual *Trichoderma* inoculation compromised the transgenic line’s resistance to *Pseudomonas syringae* and *Botrytis cinerea* compared to WT. It was previously reported that the 35S::TaEPL1-3 line accumulates elevated levels of reactive oxygen species (ROS). Therefore, ROS levels were examined to determine whether they were further influenced by inoculation with *Trichoderma* species. Dual inoculation triggered higher H_2_O_2_ accumulation in 35S::TaEPL1-3 compared to WT. In addition, high ROS levels were observed when the 35S::TaEPL1-3 line was co-inoculated with both *Trichoderma* species and subsequently challenged with both pathogens. These findings showed that elevated ROS levels may compromise priming activation in the 35S::TaEPL1-3 line (constitutively expressing the Epl1 elicitor) during co-inoculation with *T. atroviride* (Epl1-secreting) and *T. virens* (Sm1-secreting), where synergistic elicitor accumulation could potentially lead to defense signal dysregulation and consequent loss of resistance in transgenic plants.

## 1. Introduction

Plant–microbe interactions have been essential drivers of plant evolution, playing a crucial role in the colonization of terrestrial environments and the development of defense mechanisms. Throughout time, plants have established associations with a wide variety of microorganisms, including pathogens and symbionts, which significantly influence their growth, survival, and adaptation [1]. These interactions have required a coevolutionary process in which both plants and microbes have developed complex and specialized communication mechanisms to establish either beneficial or antagonistic relationships [2].

The plant microbiome comprises diverse microbial communities, including beneficial bacteria and fungi, which colonize and interact closely with their host plants. These microbes play crucial roles in promoting plant growth, facilitating nutrient uptake, enhancing tolerance to abiotic stress, and activating defense mechanisms, thereby contributing significantly to plant health and environmental adaptation [3].

Within the plant microbiome, some *Trichoderma* species, filamentous fungi capable of reducing soil-borne diseases, can establish symbiotic interactions with plants, providing various benefits such as enhanced growth, increased tolerance to both biotic and abiotic stresses, and higher seed germination rates, even in species with physiological dormancy, such as *Opuntia* [4,5,6,7]. To establish symbiotic relationships with plants, *Trichoderma* species utilize a diverse array of effector and elicitor molecules, including secreted proteins, secondary metabolites such as volatile organic compounds (VOCs) and peptaibols, as well as small RNAs. These molecules function synergistically to modulate plant defense responses, and some have also been reported to promote plant growth [8,9,10,11]. Among the secreted proteins are elicitors such as cerato-platanin proteins, which are known for their ability to trigger robust defense responses against a wide range of pathogens [12], and have more recently been implicated in the stimulation of plant growth [10].

Cerato-platanins are small, cysteine-rich proteins that appear to be restricted to filamentous fungi, with no known homologs in yeast or other eukaryotic lineages. They typically consist of 100 to 130 amino acid residues and possess a signal peptide that facilitates their secretion [13]. These proteins are secreted into the culture filtrate but are usually also present in the fungal cell wall [14]. This dual localization suggests that cerato-platanins are involved not only in fungal growth and development but also in interactions with host plants. Notably, these proteins play pivotal roles in fungus–plant interactions, functioning as elicitors in beneficial fungi and as virulence factors in pathogenic species [15,16]. The expression of cerato-platanin from *T. virens* and *T. harzianum* was induced when co-cultivated with plant roots, indicating that the plant–fungus interaction stimulates the accumulation of these elicitors [12,17]. On the other hand, cerato-platanin mutants of *Magnaporthe grisea* and *Botrytis cinerea* exhibited reduced virulence on their respective host plants [18,19].

Although the ability of the Epl1 and Sm1 elicitors from *T. atroviride* and *T. virens*, respectively, to activate plant defense responses has been reported [12,20], the specific molecular mechanisms by which these proteins exert their effects remain poorly understood. Vargas et al. [21] proposed that the monomeric forms of Sm1 and Epl1 are primarily responsible for triggering plant defenses, as these proteins can exist in both monomeric and dimeric states. Moreover, overexpression of the effector proteins Epl1 in *T. atroviride* and Sm1 in *T. virens* significantly enhanced biocontrol activity against the fungal pathogens *Alternaria solani and B. cinerea* in tomato plants, demonstrating strain-specific protective effects [22].

In a recent study, *Arabidopsis thaliana* lines expressing the *Epl1* gene from *T. atroviride* (35S::TaEPL1) were generated [10]. These engineered plants exhibited accelerated growth, suggesting that EPL1, a fungal elicitor, can act as a growth-promoting factor [10]. Moreover, TaEPL1 expression in *Arabidopsis* activated defense responses against pathogens such as *Pseudomonas syringae* and *B. cinerea* by stimulating the salicylic acid (SA) and jasmonic acid/ethylene (JA/ET) signaling pathways. Pathogen resistance in 35S::TaEPL1 lines was associated with elevated ROS levels, indicating that EPL1 expression enhances the ROS accumulation required to establish a primed defense state against pathogens. Notably, this accumulation of H_2_O_2_ was observed even in the absence of pathogen inoculation, without inducing any adverse phenotypic effects in EPL1-expressing plants [10]. While the constitutive expression of fungal elicitors such as EPL1 can enhance disease resistance in plants, as shown in the 35S::TaEPL1-3 line [10], the effects of pre-inoculating this transgenic line with two *Trichoderma* species, one producing the same elicitor and the other its homolog, on plant growth and pathogen response remain unexplored. In this study, the impact of fungal colonization on plant growth was evaluated in the transgenic *A. thaliana* line constitutively expressing the TaEPL1 elicitor. To test this, 35S::TaEPL1-3 plantlets were grown in vitro on MS medium and inoculated with *T. atroviride* and *T. virens*. In addition, the effects of single and double inoculation with *T. atroviride* and *T. virens* on pathogen resistance were evaluated in both the transgenic line and parental Col-0 plants.

## 2. Results

### 2.1. Effect of T. atroviride and T. virens Inoculation on the Growth of 35S::TaEPL1-3 Plantlets Grown In Vitro

**Figure 1:**
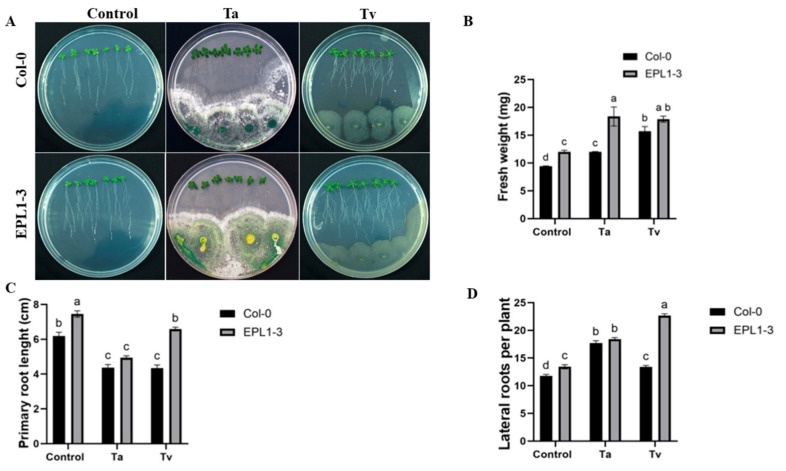
Effect of *Trichoderma* inoculation on the growth of *Arabidopsis thaliana* 35S::TaEPL1-3 line. (**A**) Representative images of 17-day-old *Arabidopsis* 35S::TaEPL1-3 transgenic and WT (Col-0) plantlets grown on MS medium, including 4 days of interaction with *T. atroviride* (Ta) or *T. virens* (Tv). Plants were inoculated at 13 days post-germination. (**B**) Fresh weight (mg) (*n* = 3), (**C**) Primary root length (cm) (*n* = 21). (**D**) Number of lateral roots per plant (*n* = 21). Data are expressed as means ± SE. Different letters indicate statistically significant differences between genotypes and treatments (two-way ANOVA with Tukey’s test, *p* < 0.05).

To determine whether fungal colonization affects the growth of transgenic *A. thaliana* plantlets expressing the TaEPL1 elicitor, 13-day-old 35S::TaEPL1-3 and the parental Col-0 (WT) plantlets were grown in vitro for 4 days in the presence of *T. virens* and *T. atroviride* spores, as well as under control (uninoculated) conditions (Figure 1A). Consistent with previous findings [10], uninoculated 35S::TaEPL1-3 plantlets exhibited higher fresh weight than Col-0 plantlets (Figure 1B). Inoculation with *T. atroviride* further enhanced this growth phenotype in the transgenic line, yielding even greater fresh weight accumulation compared to inoculated Col-0 plants. Although *T. virens*-inoculated 35S::TaEPL1-3 plantlets showed increased fresh weight compared to their uninoculated controls, their growth response did not differ significantly from *T. virens*-treated Col-0 plants. The primary root inhibition phenotype previously reported [7] was observed in 35S::TaEPL1-3 plantlets inoculated with *T. atroviride*, as well as in Col-0 plantlets inoculated with either *T. atroviride* or *T. virens*. Notably, 35S::TaEPL1-3 plantlets inoculated with *T. virens* did not exhibit this inhibition (Figure 1C). In both 35S::TaEPL1-3 and Col-0 plantlets, the number of lateral roots increased following inoculation with either *Trichoderma* species. Notably, *T. virens* treatment resulted in the highest lateral root production in 35S::TaEPL1-3 plants (Figure 1D). The 35S::TaEPL1-3 line exhibits a distinct response when interacting with *T. atroviride*, which secretes the EPL1 elicitor, compared to its interaction with *T. virens*, which secretes the orthologous SM1 elicitor and promotes longer primary roots and a greater number of lateral roots in the transgenic line.

### 2.2. The Arabidopsis 35S::TaEPL1-3 Line Exhibits Reduced Resistance to Pseudomonas syringae When Co-Inoculated with Trichoderma Species

**Figure 2:**
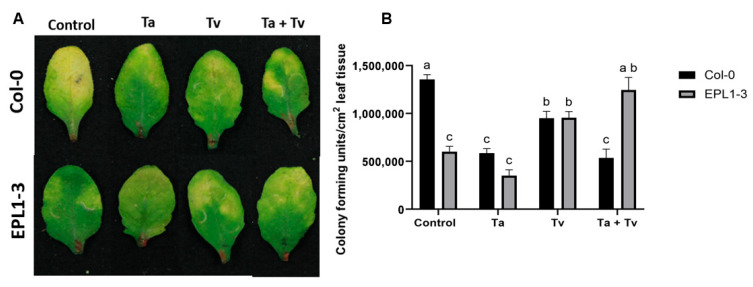
Disease symptoms caused by *Pseudomonas syringae* in *Arabidopsis thaliana* 35S::TaEPL1-3 transgenic and WT (Col-0) plants previously treated with *Trichoderma atroviride* and *T. virens.* (**A**) Representative images of *P. syringae*-infected leaves from 35S::TaEPL1-3 transgenic and Col-0 plants after *Trichoderma* pre-inoculation treatments with *T. atroviride* (Ta), *T. virens* (Tv), their combination (Ta + Tv), or no *Trichoderma* (control). Fifteen-day-old plantlets were pre-inoculated with *Trichoderma* species and subsequently challenged with *P. syringae* at 28 days. Disease symptoms were documented at 3 dpi. (**B**) Bacterial colonization quantified as colony-forming units per cm^2^ leaf area (CFU/cm^2^). Data represent mean values ± standard error (SE; *n* = 10). Different letters indicate statistically significant differences between groups (two-way ANOVA with Tukey’s test, *p* < 0.05).

The effects of single and combined pre-inoculation with *T. atroviride* and *T. virens* on the resistance to *P. syringae* were assessed in both the *Arabidopsis* 35S::TaEPL1-3 line and Col-0 (WT) plants (Figure 2A). The 35S::TaEPL1-3 and Col-0 lines were evaluated under four conditions: uninoculated control, inoculation with *T. atroviride* (Ta), inoculation with *T. virens* (Tv), and combined inoculation with both *T. atroviride* and *T. virens* (Ta + Tv). *Trichoderma* was applied when the plants were 15 days old, and bacterial inoculation was performed on the leaves of 28-day-old plants. After 3 days post-inoculation (dpi) with *P. syringae*, both uninoculated and *T. atroviride*-inoculated 35S::TaEPL1-3 plants exhibited significantly lower bacterial CFU counts compared to Col-0 plants without prior fungal inoculation (Figure 2B). This suppression of bacterial growth was similar to that observed in *T. atroviride*-treated Col-0 plants, suggesting that the transgenic line maintained its pathogen resistance despite beneficial fungal colonization (Figure 2B). Quantification of *P. syringae* growth revealed no significant difference between *T. virens*-treated 35S::TaEPL1-3 and Col-0 plants; however, both treatments resulted in lower CFU counts compared to uninoculated Col-0 controls (Figure 2). Notably, dual inoculation with both *Trichoderma* species (Ta + Tv) abolished the enhanced resistance phenotype of 35S::TaEPL1-3 plants against *P. syringae*, resulting in bacterial titers comparable to those of uninoculated Col-0 controls. Remarkably, this effect was genotype-specific, as WT (Col-0) plants colonized by the same *Trichoderma* mixture maintained robust pathogen restriction, with significantly lower CFU counts than the transgenic line lacking fungal inoculation (Figure 2B).

### 2.3. Co-Inoculation with Trichoderma Species Also Compromises the Resistance of the Arabidopsis 35S::TaEPL1-3 Line to Botrytis cinerea

**Figure 3:**
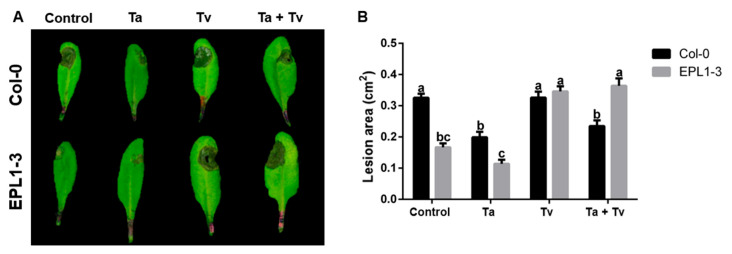
Disease symptoms caused by *Botrytis cinerea* in *Arabidopsis thaliana* 35S::TaEPL1-3 transgenic and WT (Col-0) plants previously treated with *Trichoderma atroviride* and *T. virens*. (**A**) Representative images of *B. cinerea*-infected leaves from 35S::TaEPL1-3 transgenic and Col-0 plants after *Trichoderma* pre-inoculation treatments with *T. atroviride* (Ta), *T. virens* (Tv), their combination (Ta + Tv), or no *Trichoderma* (control). Fifteen-day-old plantlets were pre-inoculated with *Trichoderma* species and subsequently infected with *B. cinerea* at 28 days. Disease symptoms were documented 3 days post-infection. (**B**) Lesion area measurements (mm^2^) of infected leaves. Data are presented as means ± SE (*n* = 20). Different lowercase letters indicate statistically significant differences between groups (*p* < 0.05, two-way ANOVA followed by Tukey’s test).

The response of the 35S::TaEPL1-3 line to *B. cinerea* infection was further analyzed following pre-inoculation with both *Trichoderma* species. In this experiment, 15-day-old plants were inoculated with *Trichoderma* species, and subsequently infected with *B. cinerea* at 28 days of age. Four conditions were evaluated: untreated controls, *T. atroviride* (Ta) pre-inoculation, *T. virens* (Tv) pre-inoculation, and dual pre-inoculation (Ta + Tv). The lesion area was observed 72 hpi with *B. cinerea* (Figure 3A). The 35S::TaEPL1-3 plants pre-inoculated with *T. atroviride* also showed a smaller infection area than Col-0 plants treated with *T. atroviride* (Figure 3B). In contrast, 35S::TaEPL1-3 and Col-0 plants inoculated with T. virens exhibited large *B. cinerea* infection areas, similar to Col-0 plants without prior *Trichoderma* inoculation (Figure 3B). Similar to the response observed during *P. syringae* infection, pre-inoculation of the 35S::TaEPL1-3 line with both *Trichoderma* species (Ta + Tv) resulted in extensive lesion development upon fungal challenge, resembling the infection levels observed in Col-0 (WT) plants without *Trichoderma* treatment. These findings suggest that the presence of both *Trichoderma* species compromises the enhanced resistance typically associated with the 35S::TaEPL1-3 line.

### 2.4. Trichoderma Co-Inoculation in the 35S::TaEPL1-3 Line Induces Increased Hydrogen Peroxide (H_2_O_2_) Accumulation

**Figure 4:**
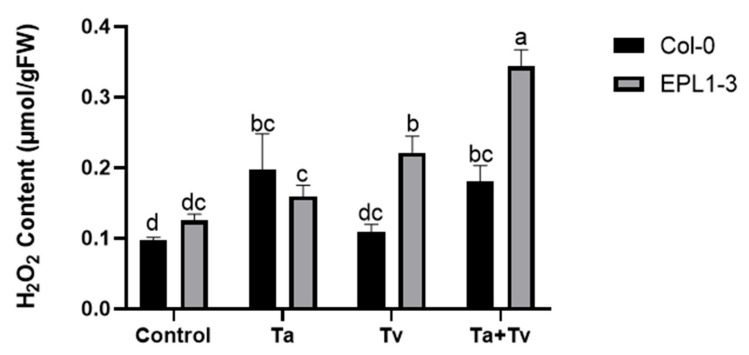
Hydrogen peroxide (H_2_O_2_) content in *Arabidopsis* 35S::TaEPL1-3 and Col-0 (WT) plants in response to *Trichoderma* inoculation. Ten-day-old plants were inoculated with *T. atroviride* (Ta), *T. virens* (Tv), or a combination of both (Ta + Tv), with non-inoculated plants used as controls. H_2_O_2_ levels were measured five days after inoculation. Data are presented as mean ± SE (*n* = 3). Different letters indicate statistically significant differences among groups (two-way ANOVA with Tukey’s test, *p* < 0.05).

The expression of the *Epl1* gene in *Arabidopsis* has been associated with increased ROS production, as shown in previous studies [10]. Based on these observations, H_2_O_2_ levels were measured in the 35S::TaEPL1-3 line and its parental Col-0 (WT) plants. Ten-day-old plants were inoculated with *T. atroviride* (Ta), *T. virens* (Tv), or a combination of both (Ta + Tv), and the H_2_O_2_ levels were measured five days later. Col-0 plantlets exhibited higher H_2_O_2_ accumulation in response to *T. atroviride* treatments and the combined inoculation with both *Trichoderma* species (Ta + Tv), compared to non-inoculated controls (Figure 4). In 35S::TaEPL1-3 plantlets, H_2_O_2_ levels increased under all fungal treatments, including individual inoculations with *T. atroviride* and *T. virens*. The highest accumulation was observed following co-inoculation with both species (Ta + Tv) (Figure 4). Notably, the highest ROS levels were detected in the transgenic line expressing the EPL1 elicitor (35S::TaEPL1-3) under co-inoculation conditions, exceeding those in its parental line Col-0. This suggests that the EPL1-expressing line elicits a stronger response when simultaneously exposed to both *Trichoderma* species.

### 2.5. Hydrogen Peroxide Content in the 35S::TaEPL1-3 Line Following Pre-Co-Inoculation with Trichoderma Species and Subsequent Pathogen Challenge

**Figure 5:**
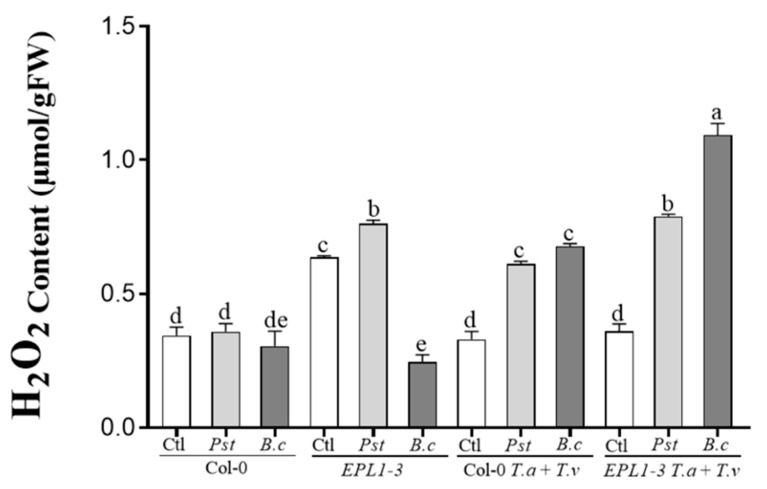
Hydrogen peroxide quantification in *A. thaliana* WT (Col-0) and 35S::TaEPL1-3 lines. Fifteen-day-old plants were first co-inoculated with *T. atroviride* and *T. virens* (Ta + Tv) or left untreated (control without *Trichoderma*), followed at 28 days by infection with either *B. cinerea* or *P. syringae.* Hydrogen peroxide levels were measured 24 h after pathogen inoculation. Data are presented as mean ± SE (*n* = 5). Different letters indicate statistically significant differences, as determined by two-way ANOVA followed by Tukey’s post hoc test (*p* ≤ 0.05).

Hydrogen peroxide (H_2_O_2_) levels were quantified in the 35S::TaEPL1-3 line and Col-0 (WT). Fifteen-day-old plants were first co-inoculated with *T. atroviride* and *T. virens* (Ta + Tv), followed at 28 days by challenge with either *B. cinerea* or *P. syringae*. Two pretreatment conditions were analyzed: untreated controls and dual inoculation (Ta + Tv). H_2_O_2_ accumulation in rosette leaves was quantified at 24 hpi with the respective pathogens. As shown in Figure 5, Col-0 plants showed higher H_2_O_2_ levels following combined *Trichoderma* pre-inoculation (Ta + Tv) compared to *Trichoderma*-uninoculated controls, for both *B. cinerea* and *P. syringae* infections. The 35S::TaEPL1-3 line showed significantly higher basal H_2_O_2_ accumulation under pathogen-free conditions compared to Col-0 controls. Following *P. syringae* infection, this line exhibited the most pronounced oxidative burst, reaching the highest ROS levels observed among all infection treatments. However, the 35S::TaEPL1-3 line did not show this enhanced oxidative response during *B. cinerea* infection, maintaining H_2_O_2_ levels comparable to *B. cinerea*-infected Col-0 plants without *Trichoderma* pre-treatment (Figure 5). Under pre-inoculation with both *Trichoderma* species (Ta + Tv), the 35S::TaEPL1-3 line showed increased H_2_O_2_ levels compared to the Col-0 (WT), both during *P. syringae* and *B. cinerea* infections, with the highest accumulation observed in response to the fungus (Figure 5). These findings imply that excessive ROS accumulation in the 35S::TaEPL1-3 line under combined *Trichoderma* pre-inoculation and pathogen challenge conditions may interfere with effective priming induction.

## 3. Discussion

Research on beneficial fungal elicitors has attracted attention due to their role in promoting plant growth and enhancing tolerance to biotic stress [9,10,23,24]. Cerato-platanins from *Trichoderma* species induce resistance by activating plant defenses, resulting in enhanced pathogen resistance [12,25].

To further understand the behavior of the *A. thaliana* 35S::TaEPL1-3 line, in vitro co-culture assays were performed with *T. atroviride* (which naturally secretes TaEpl1) and *T. virens* (which secretes TvSm1). Notably, previous work by Rojas-Moreno et al. [10] demonstrated that 35S::TaEPL1 lines displayed enhanced vigor and increased biomass accumulation relative to the parental Col-0 background. The aim was to determine whether the heterologous expression of *TaEPL1* enhances plant development during interaction with these two *Trichoderma* species. Our findings revealed that inoculation with *T. atroviride* synergistically enhanced the EPL1-3-dependent growth phenotype, resulting in significantly higher biomass accumulation in the transgenic plantlets compared to the Col-0 (WT). On the other hand, interaction with *T. virens* did not suppress primary root growth and instead promoted lateral root proliferation in the 35S::TaEPL1-3 plantlets. Taken together, these results demonstrate that the 35S::TaEPL1 line, which constitutively expresses the *EPL1* gene, displays enhanced growth compared to Col-0 plants when interacting with either *Trichoderma* species. This suggests that the expression of TaEPL1 in the 35S::TaEPL1-3 line, combined with metabolites secreted by *T. atroviride* or *T. virens*, generates a synergistic effect that enhances plant development.

The interaction between *Trichoderma* and *Arabidopsis* is complex and can significantly stimulate plant immune responses [26]. In the same study by Rojas-Moreno et al. [10], it is reported that heterologous expression of the *T. atroviride* TaEPL1 gene in *Arabidopsis* confers enhanced resistance against both bacterial (*P. syringae*) and fungal (*B. cinerea*) pathogens. This broad-spectrum resistance is mediated by salicylic acid (SA) signaling against *P. syringae*, and jasmonic acid/ethylene (JA/ET)-dependent pathways in response to *B. cinerea.* Diverse studies have shown that the application of SM1, purified from *T. virens*, enhances the expression of defense-related genes both locally and systemically in cotton [12]. Similarly, exogenous application of recombinant SM1 produced in *Pichia pastoris* was found to induce defense gene expression in maize plants [27].

The use of beneficial microbial consortia has emerged as a promising strategy in sustainable agriculture due to their additive and synergistic effects on both plant growth promotion and pathogen resistance [28]. Notably, several studies have demonstrated that *Trichoderma* consortia are effective in protecting crop plants against a variety of diseases [reviewed by 28]. The combined application of *T. viride* (PBP 4G, soil) and *T. harzianum* (Pusa 5SD, seed) significantly enhanced chickpea germination, growth, yield, and control of *Fusarium* wilt, showing greater efficacy than individual treatments against *Fusarium oxysporum* f. sp. *ciceris* [29].

In the present study, it was examined how pre-inoculation with *T. atroviride* and/or *T. virens* modulates the resistance of *Arabidopsis* 35S::TaEPL1-3 to the bacterial pathogen *P. syringae*. The findings demonstrate that both the plant genetic background and the specific *Trichoderma* species involved, or their combination, determine the plant’s resistance response to the pathogen. It was confirmed that the 35S::TaEPL1-3 line maintains enhanced resistance to *P. syringae* even without beneficial fungal colonization. Notably, this level of protection was equivalent to that observed in Col-0 plants pretreated with *T. atroviride*, suggesting that the constitutive expression of TaEPL1 in the transgenic line activates the plant immune system with comparable strength to the defense priming typically triggered by *Trichoderma* colonization. This supports the hypothesis that EPL1 functions as an effective elicitor capable of activating plant defense signaling pathways, particularly those mediated by salicylic acid, as reported by Rojas-Moreno et al. [10], which are critical for resistance against hemibiotrophic pathogens such as *P. syringae.* Pre-inoculation with *T. virens* alone did not significantly enhance resistance in 35S::TaEPL1-3 plants compared to Col-0 controls, although both lines exhibited reduced *P. syringae* titers relative to non-inoculated Col-0 plants. This suggests that *T. virens* induces defense mechanisms distinct from those activated by *T. atroviride*. Notably, the 35S::TaEPL1-3 line lost its enhanced resistance to *P. syringae* when pre-inoculated with a combination of *T. atroviride* and *T. virens*, resulting in CFU levels comparable to those observed in Col-0 plants without fungal treatment. This observation suggests a potential antagonistic interaction between the two *Trichoderma* species, which may disrupt the defense signaling pathways normally activated by Epl1 overexpression in *Arabidopsis*.

The resistance of the 35S::TaEPL1-3 line to *B. cinerea* was compromised when plants were pre-inoculated with both *Trichoderma* species, similar to the effect observed upon *P. syringae* infection. As reported by Rojas-Moreno et al. [10], the 35S::TaEPL1-3 plants displayed fewer infection symptoms caused by the fungus than Col-0. While *T. atroviride* alone appeared to reinforce resistance, the combined treatment with *T. atroviride* and *T. virens* (Ta + Tv) resulted in lesion areas comparable to those of untreated Col-0 plants. Interestingly, this same combination (Ta + Tv) in Col-0 plants promoted resistance against the fungus. These results suggest that co-inoculation may interfere with the defense mechanisms activated by EPL1 overexpression in the 35S::TaEPL1-3 line, likely due to signaling interference between the elicitors Epl1 (from *T. atroviride*) and Sm1 (from *T. virens*), further influenced by the transgenic context in which Epl1 is constitutively expressed.

Rojas-Moreno et al. [10] showed that the 35S::TaEPL1-3 line accumulated higher levels of H_2_O_2_, a response that may facilitate the rapid initiation of defense mechanisms upon pathogen challenge. In this study, H_2_O_2_ concentration was quantified using two approaches. The first involved 10-day-old seedlings exposed to five days of interaction with beneficial *Trichoderma* strains, either individually or in combination, in the absence of pathogens. The higher accumulation of H_2_O_2_ observed in the 35S::TaEPL1-3 line, particularly under co-inoculation with *T. atroviride* and *T. virens*, suggests that expression of the EPL1 elicitor gene enhances ROS production as part of the plant’s defensive response. This is consistent with previous reports linking EPL1 to the induction of ROS [10], a key component in the signaling and activation of plant defense mechanisms. Previous studies have suggested that the plant-protective effects of cerato-platanin proteins may be mediated by their capacity to trigger the accumulation of ROS and phytoalexins, thereby activating defense responses against pathogens [30,31,32]. However, this H_2_O_2_-mediated response appears to be exacerbated by co-inoculation, which could reflect a synergistic or amplified effect of the molecular signals generated by both *Trichoderma* species. The elevated endogenous levels of EPL1 in the 35S::TaEPL1-3 line, combined with the secretion of Epl1 by *T. atroviride* and Sm1 by *T. virens*, may lead the transgenic line to perceive an overaccumulation of elicitors, resulting in increased H_2_O_2_ production.

For the second experiment, H_2_O_2_ production was assessed in 35S::TaEPL1-3 transgenic and Col-0 plants. The plants, initially co-inoculated with *T. atroviride* and *T. virens* (Ta + Tv) at 15 days old, were later infected with either *B. cinerea* or *P. syringae* at 28 days. Consistent with the previous experiment without pathogens, the data showed that under pre-inoculation with both *Trichoderma* species, the 35S::TaEPL1-3 line exhibited increased H_2_O_2_ levels compared to Col-0 plants during infections with *P. syringae* and *B. cinerea*, with the highest accumulation observed in response to the fungus. These findings demonstrate that overaccumulation of ROS in 35S::TaEPL1-3 plants subjected to both *Trichoderma* pre-inoculation and pathogen attack may negatively affect priming efficiency.

Future research is needed to understand how elicitors from different *Trichoderma* species interact synergistically within plants to coordinate defense responses. In particular, studies on the 35S::TaEPL1-3 transgenic line, which expresses the *T. atroviride* Epl1 elicitor, will be key to uncovering the mechanisms activated by cerato-platanins and their potential to enhance plant immunity.

## 4. Materials and Methods

### 4.1. Plant Materials and Growth Conditions

The *Arabidopsis thaliana* lines used in this study included the transgenic line 35S::TaEPL1-3 [10] and its parental line Col-0 (WT). Seeds were surface-sterilized with a 20% (*v*/*v*) solution of commercial sodium hypochlorite (containing 6% active chlorine) for 5 min, followed by five washes with sterile distilled water. After sterilization, seeds were stratified at 4 °C for 48 h, and subsequently sown on agar plates containing 0.2× Murashige and Skoog (MS) medium (Phytotechnology Laboratories, Kansas, USA), adjusted to pH 7.0, and supplemented with 0.5% (*w*/*v*) sucrose and 0.8% (*w*/*v*) agar. Seedlings were grown in a controlled-environment growth chamber under a 16-h light/8-h dark photoperiod at 22 ± 1 °C, with a light intensity of 120 μmol m^−2^ s^−1^, for the duration specified in each experiment described below.

### 4.2. Trichoderma Growth and Inoculum Preparation

The fungal strains *Trichoderma atroviride* (IMI 206040; Ta) and *T. virens* (Gv29-8; Tv) were used to study their interaction with the 35S::TaEPL1-3 transgenic line and the WT (Col-0) *Arabidopsis* plants. Both strains were grown on Potato Dextrose Agar (PDA; Difco, NJ, USA) plates for two weeks at 28 °C. Following incubation, spores were harvested by adding sterile distilled water to the plates. Conidia were quantified using a Neubauer counting chamber under 40× magnification with a Zeiss Axioscope microscope (Carl Zeiss AG, Oberkochen, Germany). Spore suspensions were adjusted to a final concentration of 1 × 10^6^ spores/mL and used in the experiments described below.

### 4.3. In Vitro Growth Assays of Arabidopsis 35S::TaEPL1-3 and WT Lines During Interaction with Trichoderma virens and Trichoderma atroviride

To evaluate the growth response of the *Arabidopsis* 35S::TaEPL1-3 transgenic line during interaction with *Trichoderma* species, Col-0 plants were included as a control. Thirteen-day-old transgenic and Col-0 plantlets were transferred to Petri dishes containing 0.2× MS medium (seven plantlets per plate) and subjected to the following treatments: (I) control (non-inoculated plantlets), (II) inoculation with *T. atroviride*, and (III) inoculation with *T. virens*. In both inoculation treatments, 10 µL of a 1 × 10^6^ spores/mL suspension was applied to the bottom of the plate. Three biological replicates (plates) were used per treatment. Plates were incubated at 22 ± 1 °C and analyzed 4 days post-inoculation (dpi). For statistical analysis, fresh weight was averaged per plate (*n* = 3), whereas primary root length and the number of lateral roots were measured in individual plantlets (*n* = 21).

### 4.4. Pathogen Inoculum Preparation

*Pseudomonas syringae* pv. *tomato* DC3000 (*Pst*) was grown on King’s B (KB) agar medium (pH 7.0) supplemented with 50 µg/mL rifampicin and solidified with 1.5% (*w*/*v*) agar. Plates were incubated at 28 °C for 12 h. Bacterial cells were collected by adding 2 mL of 10 mM MgCl_2_ (pH 7.0) to the plate and gently resuspending the colonies. One milliliter of the resulting suspension was transferred to a microcentrifuge tube and washed three times with 1 mL of 10 mM MgCl_2_ (pH 7.0). The final bacterial suspension was adjusted to an OD_600_ of 0.04 and used to inoculate *Arabidopsis* plants as described below. To prepare the *Botrytis cinerea* inoculum, the B05.10 strain was grown on Potato Dextrose Agar (PDA; Difco) in complete darkness at 22 ± 1 °C for ten days. Spores were collected using sterile distilled water and filtered through glass wool to remove hyphal fragments. Spore concentration was determined using a Neubauer counting chamber under a Zeiss Axioscope microscope (Carl Zeiss AG, Oberkochen, Germany) at 40× magnification. The suspension was then diluted in Potato Dextrose Broth (PDB; 6 g/L) to a final concentration of 1 × 10^5^ spores/mL.

### 4.5. Determination of Pseudomonas syringae CFU in Arabidopsis 35S::TaEPL1-3 and Col-0 Plants Pre-Inoculated with Trichoderma Species

Twelve-day-old 35S::TaEPL1-3 and Col-0 (WT) seedlings, previously grown in vitro, were transplanted into 50-cell trays (4.5 cm cell diameter) containing a sterile substrate mixture composed of Sunshine Mix #3, vermiculite, and perlite in a 3:1:1 ratio. Four treatment conditions were evaluated: (I) control plants without *Trichoderma* inoculation, (II) plants inoculated with *T. atroviride*, (III) plants inoculated with *T. virens*, and (IV) plants inoculated with a mixture of both *Trichoderma* species. For treatments II–IV, 5 mL of inoculum at a final concentration of 2.5 × 10^6^ spores was applied to the substrate three days after transplantation. Starting at 15 days of age, plants were watered every three days. At 28 days old, 25 plants from each treatment group were inoculated with *P. syringae*. Three leaves per plant were infiltrated on the abaxial side with a *P. syringae* suspension (OD_600_ = 0.04) in 10 mM MgCl_2_, as described by Katagiri et al. [33]. Mock controls were infiltrated with 10 mM MgCl_2_. After inoculation, plants were covered with a transparent plastic dome to maintain high humidity and placed in a growth chamber under a 16 h light (100 μmol m^−2^ s^−1^)/8 h dark photoperiod at 22 ± 1 °C. Infection symptoms were evaluated 72 h post-inoculation (hpi). To quantify bacterial growth, six 0.5 cm^2^ leaf discs per plant were collected (*n* = 10), surface-sterilized in 70% ethanol for 1 min, rinsed with sterile water, and homogenized in 10 mM MgCl_2_. Serial dilutions were prepared, and 10 μL of a 1:5 dilution was plated on King’s B (KB) agar containing 50 µg/mL rifampicin. Plates were incubated at 28 °C in the dark for 24 h, and colony-forming units (CFUs) were counted. The assay was independently repeated at least twice, with reproducible results.

### 4.6. Quantification of Botrytis cinerea Lesion Size in Arabidopsis 35S::TaEPL1-3 and Col-0 Plants Pre-Treated with Trichoderma Species

Twelve-day-old 35S::TaEPL1-3 and Col-0 (WT) plantlets, previously grown in vitro, were transplanted into 50-cell trays (4.5 cm cell diameter) filled with a sterile substrate mixture composed of Sunshine Mix #3, vermiculite, and perlite in a 3:1:1 ratio. Plants were watered every three days throughout the experiment. Four interaction conditions were evaluated: (I) control plants without *Trichoderma* inoculation, (II) plants inoculated with *T. atroviride*, (III) plants inoculated with *T. virens*, and (IV) plants inoculated with a mixture of both *Trichoderma* species. For treatments II–IV, each plant received 5 mL of inoculum at a final concentration of 2.5 × 10^6^ spores, applied three days after transplantation. At 28 days of age, 35S::TaEPL1-3 and Col-0 plants (20 plants per line) were inoculated with a *B. cinerea* spore suspension prepared in Vogel’s buffer (sucrose, trisodium citrate·2H_2_O, K_2_HPO_4_, MgSO_4_·7H_2_O, CaCl_2_·2H_2_O, and NH_4_NO_3_). Three leaves per plant were inoculated on the adaxial surface with 10 μL drops of a *B. cinerea* spore suspension (1 × 10^5^ spores/ml), avoiding vascular tissues, following the method described by [34]. Vogel buffer was used as a mock control. After inoculation, plants were covered with a transparent plastic dome to maintain high humidity and placed in a growth chamber under a 16 h light (100 μmol m^−2^ s^−1^)/8 h dark cycle at 22 ± 1 °C. At 72 hpi, leaves were excised and photographed. Lesion areas (*n* = 20) were quantified using ImageJ (version IJ 1.54j). The assay was independently repeated twice with consistent results.

### 4.7. Determination of Hydrogen Peroxide Content Using Potassium Iodide (KI) in Arabidopsis 35S::TaEPL1-3 and Col-0

Hydrogen peroxide (H_2_O_2_) content was quantified in *A. thaliana* 35S::TaEPL1-3 and Col-0 (WT) plants at two developmental stages. In the first assay, seeds from the 35S::TaEPL1-3 and Col-0 lines were germinated in vitro on 0.2× MS medium. At 10 days of age, seven plantlets per plate were inoculated with either *T. atroviride*, *T. virens*, or a mixture of both species, and incubated for 5 days. At 15 days of age, plantlets were harvested for H_2_O_2_ quantification. Three biological replicates were used per line (*n* = 3). The second assay was conducted on 28-day-old 35S::TaEPL1-3 and Col-0 plants grown in soil and infected with *P. syringae* or *B. cinerea*. Twelve-day-old plantlets, previously grown in vitro, were transplanted into 50-cell trays (4.5 cm diameter) filled with a sterile mixture of Sunshine Mix #3, vermiculite, and perlite (3:1:1) and watered every three days. Two conditions were evaluated: (I) control plants without *Trichoderma*, and (II) plants inoculated with a mixture of *T. atroviride* and *T. virens* (5 mL per plant at 2.5 × 10^6^ spores), applied three days after transplantation. At 28 days of age, plants were infected with *P. syringae* or *B. cinerea* as described above. Infected leaves were collected 24 hpi (*n* = 5). H_2_O_2_ content in 15- and 28-day-old plants (first and second assays) was quantified following the method of Jungle et al. [35]. For each sample, 100 mg of tissue (control and treated) was homogenized on ice in 375 μL of 0.1% (*w*/*v*) trichloroacetic acid. The homogenate was centrifuged at 7000 rpm for 20 min at 4 °C. Then, 250 μL of the supernatant was mixed with 250 μL of 10 mM potassium phosphate buffer (pH 7.0) and 500 μL of 1 M potassium iodide (KI). Absorbance was measured at 390 nm using a Spectronic Genesys 10 Bio spectrophotometer (Thermo Scientific, Waltham, MA, USA). H_2_O_2_ levels were calculated using a standard curve (10–100 μM). The assay was repeated twice with consistent results.

### 4.8. Statistical Analysis

Results from representative experiments are presented as means ± SE. Statistical significance (*p* ≤ 0.05) among groups was assessed by Two-Way ANOVA (genotype × inoculation), depending on the experimental design. Tukey’s post hoc test was applied for multiple comparisons using GraphPad Prism version 10.2.2 (GraphPad Software, San Diego, CA, USA).

## 5. Conclusions

In 35S::TaEPL1-3 plants, co-inoculation with *Trichoderma atroviride* and *T. virens* abolished the enhanced resistance phenotype against *Pseudomonas syringae* and *Botrytis cinerea*. This loss of priming contrasts with WT plants, which maintained effective defense responses against pathogens following *Trichoderma* colonization. Constitutive expression of EPL1, upon exposure to dual *Trichoderma* signals, led to increased ROS accumulation. Although ROS typically function as key defense signaling mediators, their excessive accumulation in 35S::TaEPL1-3 plants correlated with diminished pathogen resistance prior to dual *Trichoderma* inoculation. This study highlights the importance of signal integration in plant–microbe interactions and offers new insights into the complexity of priming mechanisms in transgenic plants overexpressing a fungal elicitor.

## Data Availability

Data are contained within the article.
